# Bupivacaine and morphine epidural use for refractory neuropathic pain relief in a Guillain–Barré syndrome patient

**DOI:** 10.1002/ccr3.7221

**Published:** 2023-04-22

**Authors:** Eudiet Trollip, Ken Hawkins, Isabel Kwek, Krista Dewart, Mehvash Qureshi, Janek Manoj Senaratne

**Affiliations:** ^1^ Department of Anesthesiology and Pain Medicine University of Alberta Edmonton Alberta Canada; ^2^ Department of Critical Care Medicine Grey Nuns Hospital Edmonton Alberta Canada; ^3^ Department of Critical Care Medicine University of Alberta Edmonton Alberta Canada; ^4^ Department of Medicine, Division of Cardiology University of Alberta Edmonton Alberta Canada

**Keywords:** epidural, Guillain–Barré syndrome, neuropathy, pain

## Abstract

Pain is common in the acute phase of Guillain–Barré Syndrome and can be severe and refractory. Pain in GBS may not always respond to contemporary pain therapy. An epidural can potentially be considered for the treatment of refractory pain after a careful patient‐centered discussion with the patient about risks.

## 1 BACKGROUND

Pain is common in the acute phase of Guillain–Barré syndrome (GBS) and can be severe and refractory. Pain in GBS does not always resond to contemporary pain therapy. An epidural can potentially be considered for the treatment of refractory pain after a careful patient‐centered discussion with the patient about risks. Contemporary data on the use of epidurals for refractory pain management in GBS are lacking. This case report describes the use of an epidural to manage severe refractory pain in a patient with GBS.

GBS is an immune‐mediated disease of the peripheral nerves and nerve roots that are usually triggered by infection. It is characterized by acute progressive motor weakness and areflexia. Pain is a relatively common symptom in GBS. The reported frequency of pain is variable (55%‐89%) and can range in intensity from very mild to severe.^1^ Younger GBS patients are especially susceptible to pain, which can cause both prolonged hospital stay and recovery, as well as long‐term disability. Pain can be either neuralgic or myalgic in nature. There are little data on the optimum medical treatment of pain but pharmacologic agents that are typically used and studied include acetaminophen, nonsteroidal anti‐ inflammatory drugs, gabapentin, opioids, ketamine, lidocaine, tricyclic antidepressants, and selective serotonin reuptake inhibitors.^2^ In one study, pain was reported in 36% of patients in the 2 weeks preceding weakness, 66% during the acute phase, and 38% still reported pain after 1 year.^3^ Severe refractory pain is relatively uncommon but does complicate the course of certain patients. Very little data are available on the treatment of pain refractory to traditional and adjunct oral/intravenous medications.^4^ In the mid‐1980s, there were a few published case reports on the use of epidurals for GBS pain.^5^ There have not been any published case reports or studies in the last 25 years. Given that refractory pain in GBS is relatively rare, there are no randomized controlled trials on the pain management of this difficult to treat subset of patients with GBS.^6^ In this case report, we describe the use of an epidural in a patient with GBS with refractory pain syndrome. An epidural infusion of morphine and bupivacaine was started at 10 mL/h, and after 6 h, the patient's pain went from a Critical Care Pain Observation Tool score (CPOT) of 8/10 to 2/10. Most adjunctive opiates were stopped within a day with the epidural being used for a pain break. No complications from the epidural trial were seen.

## 2 CASE PRESENTATION

A previously healthy male in his twenties presented initially with symptoms of chills, night sweats, a sore throat, mild cough, and upper back and abdominal pain. Over the next 10 days, symptoms progressed to painful paresthesia, and weakness in the lower limbs, which ascended and progressed to complete lower extremity paralysis, constipation, and urinary retention. A lumbar puncture done on admission to hospital showed a protein level of 1.08 g/L with a white blood cell count of 0 cells/mm3. Nerve conduction studies were undertaken as per neurology consultation and were suggestive of an acute motor greater than sensory axonal and demyelinating polyneuropathy with conduction block. The studies also suggested prolonged F waves and distal motor latency prolongation in keeping with the diagnosis of GBS. At this time, the patient was admitted to the intensive care unit and started on intravenous immunoglobulin immediately at 400 milligrams/kilogram for 5 days. Over the next 48 h, his respiratory status deteriorated from room air to 4L of oxygen per minute with decreased lung volumes on chest x‐rays. His negative inspiratory force was < 15 centimeters of water. He was electively intubated and mechanically ventilated due to the risk of acute respiratory failure.

Three days postintubation (14 days since symptom onset), the patient's pain significantly worsened (localized mainly to the lower extremities and chest), and he was started on gabapentin 900 mg daily, ibuprofen 1600 mg daily, and intravenous hydromorphone 1 mg as needed, as suggested in single‐centre, small randomized controlled trials.^7^ His pain was measured using the Critical Pain Observation Tool (CPOT) scoring scale, which includes four behavioral indicators: facial expression, body movements, muscle tension, and compliance with the ventilator. Each item is scored from 0 to 2 for a possible total score range from 0 to 8 points. His symptoms did not improve, and his CPOT remained 8/10. 18 days after admission to hospital, a tracheostomy was performed. His pain further increased, and he was only able to sleep 1 h per night with his CPOT score constantly ranging from 8/10‐10/10. On day 24, the patient was started per guidelines on venlafaxine 75 mg and zopiclone 7.5 mg.^8^ Exact drug dosages with timeline of escalation of therapy are provided in Table 1. On day 29, ketamine was started to a total of 40 mg daily via nasogastric tube followed by olanzapine 3 mg daily on day 31. Pain remained unchanged and sleep only slightly improved to 1‐2 h a night, and on day 34, nabilone 2 mg daily was started and a fentanyl patch added at 25 mcg/h. On day 35 due to continued poor pain control, a lidocaine infusion was started at 3.84 mg/h with slight improvement in pain to a CPOT of 7/10. At this point, the patient was on total daily doses of acetaminophen 3900 mg, diclofenac gel 2.32%, fentanyl patch 37.5 mcg/h, gabapentin 3600 mg, dilaudid 30 mg, ibuprofen 1600 mg, ketamine 40 mg, lidocaine infusion 3.84 mg/h, nabilone 6 mg, and olanzapine 2.5 mg and still had uncontrolled pain and could not tolerate weaning of any of the agents. He had not suffered any significant side effects of new onset headaches, nausea, blurry vision, seizures of dizziness from the medication at this time, with renal function and liver function being maintained. However, the patient's negative inspiratory force was essentially undetectable, which was likely partially affected by all the sedation.

**TABLE 1:** Analgesia dosing before, during, and after epidural use.

Medication	Beginning Dosage	Highest Dosage	Dosage After Epidural	Total Duration of Therapy (Days)
Acetaminophen (mg/day)	2600	3840	3840	39
Diclofenac gel %	2.32	2.32	‐	3
Fentanyl Patch (mcg/h)	25	37.50	38	16
Gabapentin (mg/day)	300	3600	3600	39
Dilaudid (mg/day)	7	30	10	36
Ibuprofen (mg/day)	1600	1600	‐	27
Ketamine (mg/day)	30	40	‐	9
Lidocaine Infusion (mg/day)	3840	3840	‐	7
Nabilone (mg/day)	2	6	6	16
Olanzapine (mg/day)	2.50	3	3	18
Phenytoin (mg/day)	1500	1500	‐	1
Venlafaxine (mg)	75	120	120	26
Epidural (Morphine 10‐bupivacaine 250 mg at mL/h)	10	10	‐	6
^a^Cpot Score	8	8	8	16

^a^ CPOT—Critical Pain Observation Tool.

On day 37, a family conference with the patient and family regarding epidural use in GBS was held with the potential risks and benefits discussed. They were informed of the lack of existing data to support the use of an epidural for GBS pain. At this time, the patient and his family provided informed consent to trial the epidural for a pain break. This was 39 days after initial symptom onset and 24 days after severe pain was reported by the patient. The patient agreed to a maximum of 5 days of epidural use to minimize the risk of local complications with a primary goal of a short pain break along with the psychological reassurance that his pain could be controlled. The weaning strategy postepidural was also discussed with the patient.

## 3 TREATMENT

A lumbar epidural was inserted by Anesthesia with a 9 cm Tuohy catheter, which was inserted without complication. A continuous infusion was started with Morphine 10 mg‐ Bupivacaine 250 mg in normal saline 250 mL running at 10 mL/h.

**FIGURE 1:**
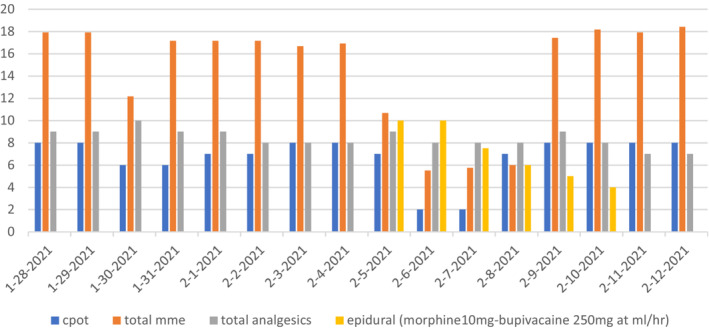
Graph showing change in pain scores and analgesic requirements based on epidural use.

One day before insertion of the epidural, the patient was on a total milligram morphine equivalent (MME) of 125 mg along with Lidocaine at 3.84 mg/h, and Gabapentin 1800 mg orally daily. Pain was still documented as 8/10 on the CPOT. A day after epidural insertion, the patient was able to tolerate an opioid reduction to MME of 75 mg along with lidocaine and ketamine discontinued with the epidural at 10 mL/h and a CPOT of 2/10 as shown in Table 2 and the graph in Figure 1. 2 days after insertion, the epidural rate was decreased to 7.5 mL/h and his total MME was further down by about 40% with hydromorphone being as low as 1 mg total daily and the fentanyl patch decreased to 12.5 mcg/h with the CPOT scores maintaining at 2/10. On day 6, after slowly decreasing the epidural rate, the epidural catheter was removed, and pain eventually increased again resulting in subsequent increases in opiates to an MME of 150 mg with a CPOT score of 8/10.

**TABLE 2:** Pain score before, after, and during epidural use.

Days since symptom onset	CPOT^a^ score	Total daily MME^b^ (mg)	Total number daily analgesics	Epidural (Morphine 10 mg – Bupivacaine 250 mg at mL/h)
34	8	17.92	9	‐
35	8	17.92	9	‐
36	6	12.17	10	‐
37	6	17.17	9	‐
38	7	17.17	9	‐
39	7	17.17	8	‐
40	8	16.67	8	‐
41	8	16.92	8	‐
42	7	10.67	9	10
43	2	5.5	8	10
44	2	5.75	8	7.5
45	7	6	8	6
46	8	17.42	9	5
47	8	18.17	8	4
48	8	17.92	7	‐
49	8	18.42	7	‐

^a^ CPOT—Critical Pain Observation Tool.

^b^ MME—Morphine Milligram Equivalents.

## 4 OUTCOME AND FOLLOW‐UP

The patient reported a significant decrease in pain after insertion of the epidural. His CPOT score went from 8/10 the day before insertion to a score of 2/10. He also tolerated the discontinuation of the lidocaine infusion, as well as a significant reduction in opioid use. As per the stated initial goals, after a pain break of a few days, the epidural was discontinued to reduce the risk of any complications with a subsequent increase in CPOT scores back to 8/10 and an increased need for opioids. The patient was subsequently decannulated on day 63 since onset of symptoms, and by day 74, his pain was much improved with a CPOT of 2/10 daily. From day 77, the patient was discharged from ICU with no side effects to the epidural, no postdural headache, no epidural abscess or hematoma, and no nerve damage. He was admitted at this point to a ward to continue rehabilitaion. He was discharged from hospital to a rehabilitation centre on day 115 needing only gabapentin 1200 mg daily, venlafaxine 75 mg daily, and nortriptyline 35 mg daily.

## 5 DISCUSSION

Pain is a common complicating factor in GBS. However, severe refractory pain is relatively uncommon but can omplicate the course of some patients with GBS. There are limited data on how to best treat these patients. Data on the use of an epidural for refractory pain relief in GBS are even further limited. The potential benefit is a reduction in refractory pain, which can have a psychological impact on patients (and families). Moreover, the use of an epidural could potentially spare the use of other medications such as opioids with their multitude of side effects and dependence, as well as the use of other medications, which may have varied effects such as QT prolongation, which in itself can increase mortality. Furthermore, most of these medications cause respiratory depression, which may prolong the time that a GBS patient is on a ventilator, which has its own risks such as deconditioning, ventilator induced diaphragm dysfunction, ventilator induced lung injury, stress ulcers, decubitus ulcers, and ventilator‐associated pneumonia. This host of side effects to traditionally used pain management plans have prompted the development of safer therapeutic modalities.^9^

The potential risks of an epidural include that of localized infection or bleeding, which may go unnoticed especially in a patient that is already paralyzed and unable to sense. However, this risk is approximately 1/18,000 for epidural hematomas and an incidence of 0.2 to 2.8 cases per 10,000 for an epidural abscess and potentially could be reduced further with daily screening ultrasounds to look for localized fluid collections.^10^, ^11^ Furthermore, a tunneled epidural could further reduce the risk of infection though evidence is lacking in GBS patients.^12^ Studies have supported the use of tunneled epidurals to provide not only timely but extended analgesia for cancer patients.^13^

In our case, the epidural produced a significant improvement in the refractory dysesthetic pain for our patient. The epidural was only kept in situ for 5 days to prevent infection.

Most case studies documenting the use of epidural after failure of routine analgesics in GBS were documented in the late 80s and 90s. All used morphine sulphate epidurals in previously healthy adults. A short report from 1991 showed that 8/9 patients benefited from a morphine epidural to treat refractory pain with minimal side effects with the most common being urinary retention and pruritis.^14^ Again, in 1992, a case study successfully reported the long‐term use of a bupivacaine and fentanyl epidural; however, after day 24, opioid requirements increased suggesting development of tachyphylaxis.^15^

Our case is novel in that more data have emerged since the 1990s in the use of adjunct therapies for pain control in GBS. In our case, our patient was already on what would be considered the contemporary management of pain in GBS with multiple adjunct agents. Even in the setting of being on all these drugs, the patient still had refractory pain. Despite this, the epidural was highly effective, which is in contrast to all previous studies from the 1980s where epidurals were used with very little adjunct medication on board.

Further data are required on epidurals in GBS. Additionally, larger, well designed randomized control trials are required to further investigate the safety of potential interventions for patients with pain in GBS.^16^ The present case report gives some observational evidence to the potential benefit of epidurals in GBS and may be a steppingstone to considering a trial of epidurals (or tunneled epidurals) in patients with GBS and refractory pain.

## AUTHOR CONTRIBUTIONS

**Eudiet Trollip:** Conceptualization; data curation; formal analysis; funding acquisition; investigation; methodology; project administration; resources; supervision; validation; writing – original draft; writing – review and editing. **Ken Hawkins:** Conceptualization; data curation; investigation; methodology; supervision; validation; writing – review and editing. **Isabel Kwek:** Conceptualization; data curation; formal analysis; investigation; methodology; resources; validation; writing – original draft; writing – review and editing. **Krista Dewart:** Conceptualization; data curation; formal analysis; investigation; methodology; validation; writing – original draft; writing – review and editing. **Mehvash Qureshi:** Conceptualization; data curation; formal analysis; investigation; methodology; validation; writing – original draft; writing – review and editing. **Janek Manoj Senaratne:** Conceptualization; data curation; formal analysis; funding acquisition; investigation; methodology; project administration; resources; supervision; validation; visualization; writing – original draft; writing – review and editing.

## FUNDING INFORMATION

None.

## CONFLICT OF INTEREST STATEMENT

I would like to report that there is no existing or potential conflict of interest.

## CONSENT STATEMENT

Written informed consent was obtained from the patient to publish this report in accordance with the journal's patient consent policy.

## Data Availability

Data sharing not applicable to this article as not datasets were generated or analyzed during the current study.
